# Long-Term Clinical Outcome of Tacrolimus Skin Ointment (0.03%) for the Treatment of Vernal Keratoconjunctivitis: A Quasi-Experimental Study

**DOI:** 10.7759/cureus.50579

**Published:** 2023-12-15

**Authors:** Warda Ali, Saad Alam khan, Fahim Ullah Khan, Shama Khan, Wajid A Khan, Rabeeah Zafar, Muhammad A Moqeet

**Affiliations:** 1 Cornea and Refractive Surgery, Al-Shifa Trust Eye Hospital, Rawalpindi, PAK

**Keywords:** topical steroid, conjunctivits, long term, tacrolimus, vernal keratoconjunctivitis

## Abstract

Background

Vernal keratoconjunctivitis (VKC) is an allergic conjunctival inflammation with severe ocular complications if left untreated. The current management regimen is plagued with adverse effects, long-term problems, and clinical relapses. Tacrolimus offers an alternative treatment option, and long-term studies are needed to determine its efficacy.

Methods

A two-year follow-up based study was conducted on moderate to severe VKC patients, who were prescribed tacrolimus skin ointment. The 5-5-5 exacerbation scale was used for the monitoring and grading severity of the disease. Analysis of variance (ANOVA) and intergroup comparisons were conducted on exacerbation scale scores among follow-ups.

Results

A significant reduction was observed in the total score of severity from baseline (203.17±102.05) to three months' follow-up (69.94±70.54), and it kept reducing for 18 months post therapy. Similar results with statistically significant reduction were observed for all grades of the scale. The relapse rate was 5.71% within a month after therapy cessation, and none of the other patients showed relapse afterward. No significant ocular and systemic complications were observed during the study.

Conclusion

Tacrolimus is effective in the long-term management of VKC without the complications of conventional steroid-based therapy.

## Introduction

Vernal keratoconjunctivitis (VKC) is an acute or chronic inflammation of the cornea and conjunctiva (tarsal and bulbar) that occurs in children and adolescents with seasonal exacerbations [1,2]. It has a higher incidence in a hot and dry climate and is common in South Asia, Central Africa, and South America. It is an important public health problem in Asia, having a significant effect on the quality of life and productivity of individuals affected by it [3].

VKC starts as a type 1 (immediate) hypersensitivity reaction in which antigen causes cross-linking of two adjacent immunoglobulin E (IgE) molecules that initiates degranulation of mast cells inducing the release of histamine leading to the classical signs of allergic reaction. Later, the disease becomes chronic due to type 2 lymphocyte (helper) invasion with activation of fibroblasts in the cornea and conjunctiva, leading to corneal and tarsal conjunctival changes. VKC must be differentiated from seasonal and other allergic conjunctivitis, which are type 1 hypersensitivity (IgE) mediated and have only conjunctival involvement in the majority of cases [2,4,5].

A variety of inflammatory mediators are released by mast cells, such as histamine, leukotrienes (IL), prostaglandins, chymase, tryptase, chondroitin sulfate, and heparin. These cause increased vascular permeability with the involvement of eosinophils, T and B lymphocytes, and fibroblasts that increase collagen deposition in the conjunctiva. IL-4 and IL-13 specifically cause the formation of conjunctival giant papillae by extracellular matrix deposition in fibroblasts [4-8].

The clinical presentation of VKC is asymmetric in most cases and is classified based on the affected area, which can be palpebral, limbal, or involving both of these components. The conjunctiva shows an increase in vessel permeability with cellular recruitment and epithelial hyperplasia. The upper tarsus is commonly involved with the formation of giant papillae with a diameter of more than 0.3 mm producing the cobblestone appearance (the hallmark of VKC). In severe VKC, papillary hypertrophy produces cauliflower-like excrescences. Furthermore, the advanced involvement of papillary conjunctiva can lead to symblepharon formation.

Limbal signs include thickening and opacification of the conjunctiva and gelatinous papillae along with a perilimbal collection of degenerated epithelial cells and eosinophils known as Horner-Trantas dots. Limbal disease can lead to limbal stem cell deficiency and pannus formation with neovascularization of the cornea. The corneal features depend upon the severity of conjunctival inflammation. The toxic effects of inflammatory mediators may lead to punctate epithelial erosions (PEEs). These PEE coalesce into macroerosions and the accumulation of mucus and fibrin into macroerosions leads to the formation of shield ulcers [9].

There are certain unmet needs regarding the management of VKC, such as the absence of diagnostic criteria, unclear pathogenesis, and ineffectiveness of the mainstay topical anti-allergic treatment (antihistamines) in cases with moderate to severe diseases. This lack of standardized diagnostic and treatment protocols leads to variations in management regimes among different countries. Moreover, safety and complications of drugs and compliance with therapy are other issues. Thus, considering the immunomodulatory role of tacrolimus, it will be beneficial in the complete control of moderate to severe VKC. After all, a satisfactory and regular treatment based on the tenets of individual patient requirements and long-term follow-up is essential to see the efficacy and safety of treatment with tacrolimus that will help in setting new guidelines in treatment protocols in the future [3,10-12].

The treatment of VKC should be according to the duration and frequency of symptoms and the severity of signs. Mild cases are improved with topical mast cell stabilizers, antihistamines, combine antihistamine mast cell stabilizers, non-steroidal anti-inflammatory drugs, and lubricants. However, severe cases frequently have remissions and relapses, require prolonged treatment, and result in visual compromise, if not properly treated [2].

Corticosteroids are added in addition to anti-allergic drugs, and they have to be used in long term to control symptoms [13]. However, steroids’ withdrawal increases disease severity, and long-term use results in cataract formation, glaucoma, and keratoconus [14]. Immunomodulators, such as cyclosporine, INFα2b, and tacrolimus, are alternatives to steroids in controlling moderate to severe symptoms. Cyclosporine 1% is given three to four times per day and is effective for seasonal exacerbations. Tacrolimus is almost 100 times more potent as compared to cyclosporin and is isolated from the fermentation broth of *Streptomyces tsukubaenis* [15,16]. INF-α2b is also equally effective as tacrolimus but has limited availability.

Tacrolimus is a calcineurin inhibitor that binds FK506-binding proteins in T-lymphocytes. This leads to a decreased activity of T cell (type 1 and 2) cytokines, such as IL-2, interferon γ, IL-4, and IL-5. Tacrolimus also has a role in inhibiting histamine release from mast cells [17]. Conjunctival cytology of patients treated with tacrolimus revealed a reduction in inflammatory cells, most commonly eosinophils [18]. Tacrolimus was first used as an immune suppressant in the liver and solid organ transplants. It has also been used for treating certain skin conditions, such as atopic dermatitis and vitiligo. Although no high-risk complications have been observed, side effects, such as ocular burning, itching, and increased sensitivity to heat and light, have been reported.

Tacrolimus has been used as effectively for the treatment of multiple conjunctival inflammatory conditions, such as VKC, atopic keratoconjunctivitis (AKC), giant papillary conjunctivitis, uveitis, Mooren’s ulcer, corneal graft rejection, blephrokeratoconjunctivitis, and chronic follicular conjunctivitis [15,19,20].

Although studies regarding the efficacy of tacrolimus have been reported in the literature, very limited work has been done regarding its long-term outcomes to the best of our knowledge. In addition, work has been done on ocular preparations of tacrolimus, which are not available in developing countries, especially Pakistan, a country with a high burden of VKC. Thus, we used 0.03% tacrolimus skin ointment and conducted a 24-month study to see its efficacy and safety in severe cases of VKC. The aim is to set the best available evidence in clinical practice and to assist in setting guidelines for the management of VKC in developing countries [21].

## Materials and methods

A prospective nonrandomized interventional study was conducted at the Department of Cornea and Refractive Surgery, Al-Shifa Trust Eye Hospital, Pakistan, from July 2020 to July 2022. The ethical approval was taken from the Ethical Review Committee of Al-Shifa Trust Eye Hospital (reference no.: ERC-67/AST-20), and all the procedures were conducted in accordance with the protocols of the Declaration of Helsinki. Patients with newly diagnosed or recurrent moderate to severe VKC were consecutively recruited into the study. The exclusion criteria included all other forms of allergic conjunctivitis, giant papillary conjunctivitis, history of subconjunctival, and systemic steroid use.

Examination protocols

All the participants on their baseline assessment were categorized by utilizing the 5-5-5 exacerbation scale (21). This system classifies all the features of VKC into three different categories (100-point scale, 10-point scale, and one-point scale) based on the severity of the disease. Baseline and follow-up examinations were done by a single clinician (WA).

Written informed consent was obtained from all participants before their inclusion in the study. A thorough medical and ocular history was obtained at the time of baseline assessment, which was followed by a complete ocular examination. An initial grading was done based on the classification system described above. Afterward, Eczemus (tacrolimus 0.03%) skin ointment (Brookes Pharma Ltd., Karachi, Pakistan) was prescribed a frequency of two times a day depending upon the severity of the disease. Eczemus has been reported to be used effectively for ocular uses in previous literature [2,22-25]. No conjunctive therapy was provided to the participants other than artificial lubricants depending upon the ocular condition. Although the frequency of treatment was not altered depending upon the changes in the severity of the disease, it was continued for 12 months. Regular follow-ups were advised for all patients at regular intervals. However, data were collected on the follow-up at one month, three months, and afterward at every six months' interval after the initiation of therapy. Lastly, the blood profile along with renal and liver function tests was assessed at every follow-up after three months of initiation of therapy. In addition, the patient was thoroughly interviewed for any other side or adverse effects, which they have experienced during this therapy.

Outcome variables

The primary outcome of the study was the severity of VKC, which was assessed by calculating the final score based on the clinical signs observed at every follow-up. In addition to the total grading, the score of every category was also recorded. The relapse rate after reduction in the frequency of therapy was also assessed, which was calculated as the ratio of cases that showed a progression in the severity of the disease, leading to a 183-point increase in the total score to the total cases in which therapy was prescribed. Lastly, frequencies of side and adverse effects experienced by the participants and their severity were also assessed as the outcome of the study.

Statistical analysis

Complete analysis of data was conducted in IBM SPSS Statistics for Windows, version 21 (released 2012; IBM Corp., Armonk, New York, United States). Rigorous data cleaning was done before the final analysis. In the descriptive analysis, frequencies were described for all categorical variables. The continuous variables were presented descriptively by utilizing the mean along the standard deviation and total ranges.

Repeated-measures analysis of variance (ANOVA) was used for the assessment of changes in scores in 5-5-5 grading with therapy. It was conducted at a confidence interval of 95%. A P value of <0.05 was considered as statistically significant.

## Results

A total of 70 eyes from 70 patients were included in the study. The mean age of the participants was 15.4±3.22 years ranging from eight to 22 years. The gender distribution showed that the majority of the individuals were male (61.40%, n= 43), and the rest were female.

On the baseline assessment, the mean score was 203.17±102.05, which initially showed a slight increase after one month (206.10±122.22) but subsequently reduced at every follow-up with a score of 9.06±28.48 (range=0-101). A similar reduction was also noted for scores pertaining to different severity levels as the mean score in the 100-point category reduced from 178.57±100.57 (0-400) to 12.85±33.71 (range=0-100) at one-year post therapy examination, which further reduced to 7.14±25.93 (range=0-100) after another year (Table 1).

**Table 1:** Score according to 5-5-5 exacerbation grading scale at baseline and follow-up (mean±standard deviation). P-value <0.05 was considered as significant.

Score	Baseline	One month	Three months	Six months	One year	Eighteen months	Two years	p-value
10 points	19.57±13.34	17.71±13.95	4.14±6.7	0.85±2.81	0.71±2.59	57±2.33	0.28±1.67	<0.001
100 points	179.71±100.84	185.50±120.38	65.21±68.22	18.84±39.39	11.59±32.25	8.69±28.38	7.24±26.11	<0.001
1 point	3.88±1.09	2.91±1.68	0.67±1.03	0.25±0.715	0.14±0.571	0.085±0.329	0.071±0.259	<0.001

**Figure 1:**
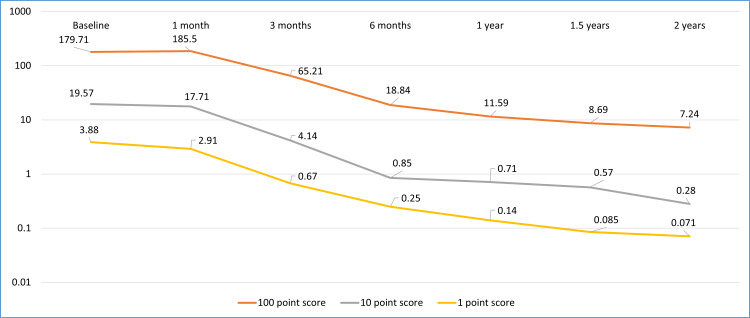
Line chart for VKC scores at different follow-ups on a logarithmic scale

It was also observed that the total mean scores of follow-up visits showed a statistically significant reduction in comparison to the baseline examination except for the visit at one month (p-value=1.00). Furthermore, a statistically significant reduction in mean scores between subsequent visits was observed until follow-up at six months, after which the stability of the scores was recorded as the difference in mean values was not statistically significant. The 10-point score along with others also showed a similar pattern of change (Table 2). This has also been shown in Figure 1 that tacrolimus equally reduced the features of VKC on every level of severity on the 5-5-5 scale.

**Table 2:** Comparison at baseline and follow-ups (standardized mean difference). P-value <0.05 was considered as significant.

Groups	Total	P-value	100-point	p-value	10-point	p-value	1-point	p-value
Baseline-Fup1	-2.928	(1)	-5.797	(1)	1.857	(1)	0.971	(<0.001)
Baseline-Fup2	133.23	(<0.001)	114.49	(<0.001)	15.42	(<0.001)	3.214	(<0.001)
Baseline-Fup3	183.36	(<0.001)	160.87	(<0.001)	18.714	(<0.001)	3.629	(<0.001)
Baseline-Fup4	190.85	(<0.001)	168.11	(<0.001)	18.857	(<0.001)	3.743	(<0.001)
Baseline-Fup5	193.89	(<0.001)	171.01	(<0.001)	19.00	(<0.001)	3.800	(<0.001)
Baseline-Fup6	194.11	(<0.001)	172.46	(<0.001)	19.28	(<0.001)	3.814	(<0.001)
Fup1-Fup2	136.15	(<0.001)	120.29	(1)	13.57	(<0.001)	2.243	(<0.001)
Fup1-Fup3	186.29	(<0.001)	166.66	(<0.001)	16.857	(<0.001)	2.657	(<0.001)
Fup1-Fup4	193.78	(<0.001)	173.91	(<0.001)	17.00	(<0.001)	2.771	(<0.001)
Fup1-Fup5	196.82	(<0.001)	176.81	(<0.001)	17.14	(<0.001)	2.82	(<0.001)
Fup1-Fup6	197.04	(<0.001)	178.26	(<0.001)	17.42	(<0.001)	2.84	(<0.001)
Fup2-Fup3	50.13	(<0.001)	46.377	(<0.001)	3.286	(<0.001)	0.414	(<0.001)
Fup2-Fup4	57.62	(<0.001)	53.623	(<0.001)	3.429	(<0.001)	0.529	(<0.001)
Fup2-Fup5	60.66	(<0.001)	56.522	(<0.001)	3.571	(<0.001)	0.586	(<0.001)
Fup2-Fup6	60.88	(<0.001)	57.97	(<0.001)	3.857	(<0.001)	0.600	(<0.001)
Fup3-FUp4	7.493	(0.416)	7.246	(0.509)	0.143	(1)	0.114	(0.218)
Fup3-Fup5	10.53	(0.110)	10.145	(0.151)	0.286	(1)	0.171	(0.083)
Fup3-Fup6	10.75	(0.093)	11.594	(0.082)	0.571	(0.938)	0.186	(0.123)
Fup4-Fup5	3.043	(1)	2.899	(1)	0.143	(1)	0.057	(1)
Fup4-Fup6	3.261	(1)	4.348	(1)	0.429	(1)	0.071	(1)
Fup5-Fup6	0.217	(1)	1.449	(1)	0.286	(1)	0.014	(1)

The complaint of a burning sensation was made by 10 patients (14.28%). None of the patients had any significant ocular and systemic complications in our study, and relapse after discontinuation of therapy was observed in 5.71% (n= 4) of the sample who were then started again on the same therapy.

## Discussion

VKC has a significant impact on the quality of life and daily activities of patients because symptoms can lead to a lack of sleep, which has an impact on outdoor activities and school dropouts. In addition, increased duration and severity of the disease have a substantial effect on the quality of life of adults and financial impact. Thus, such patients require psychological support to avoid overmedication, such as corticosteroids leading to decreased vision and under-use of the drug resulting in scarring and stem cell deficiency [3]. 

In developing countries, such as Pakistan, there is non-availability of an ocular topical preparation of tacrolimus, so considering the potential role of tacrolimus in controlling severe cases of VKC and the use of dermatological ointment in various studies [2,22-25] with similar beneficial effects as topical therapy, we also used tacrolimus skin ointment in order to prove its potent role in the stabilization of VKC.

We evaluated the clinical outcome and safety of tacrolimus skin ointment (0.03%) in patients suffering from VKC. Our results revealed three important findings. First, the 12-month therapy with tacrolimus skin ointment 0.03% showed a reduction in clinical scores from 203.17±102.05 that was 206.10±122.21 at three weeks and improved significantly to 69.94±70.54 and 19.81±40.29 at three and four months, respectively. Second, tacrolimus skin ointment 0.03% has similar effects on reduction in scores of 100-, 10-, and one-point signs. This means that the drug can be given to eyes irrespective of the signs in the exacerbation grading scale, with a linear reduction pattern of 100-, 10-, and one-point signs. Third, no serious adverse effects were noted during our study period, which means that it is safe for the treatment of VKC.

Severe VKC is considered a refractory form of ACDs, and this study would guide the treatment of such patients. This study demonstrates that a 12-month use of tacrolimus skin ointment 0.03% is effective in reducing all symptoms of VKC, including cobblestone papillae, and helps to maintain the stable stages of VKC, thereby improving the quality of life of patients.

Almost all patients in our study showed dramatic improvements in clinical scores without developing any significant adverse effects. The only adverse effect was a burning sensation, which was reported by 10 patients. Only three patients were non-responders that were shifted to topical antihistamines and steroids. There were four patients that showed recurrence of disease one month after cessation of the tacrolimus therapy, which again responded when tacrolimus was started. In the study by Hirota et al., there was an increased remission rate at the 24th month with prolonged use of tacrolimus ophthalmic suspension (0.1%) [26-28]. This suggests that to achieve maximum response with therapy, it should be continued for two consecutive seasons in patients with severe symptoms. Considering the stable stages of VKC, further studies are needed to detect the role of the proactive immunosuppressant drug in low doses to prevent the recurrence of the disease. The effectiveness and safety of topical tacrolimus 0.1% over six months has been evaluated in a nationwide survey in Japan and included more than 1,000 patients with VKC and AKC, but topical steroids were given when needed [29]. Yazu et al. reported long-term improvement in clinical signs of severe and refractory VKC and AKC with tacrolimus, but the sample size was small [30].

The adverse effects of tacrolimus noted in our study and the literature are burning and stinging. Reactivation of herpes simplex viral keratitis was a concern because of the immunosuppressive effect of tacrolimus [20], but none was reported in our study and the literature.

A recent meta-analysis included five studies and reported significantly lower ocular objective sign scores (standardized mean difference (SMD) −1.39, 95% CI −2.50 to −0.27; p < 0.05 and subjective symptom evaluation score (SMD −0.92, 95% CI −1.59 to −0.24; p< 0.05). There was high heterogeneity in this study because the control was not the same. Some research groups were given a placebo, while others were given cyclosporin, interferon α-2b, or tobramycin dexamethasone. The subjective symptom scores of the patients in the tacrolimus trial group (TAC) at the end of the treatment were significantly lower than those of the control group. There was a difference in drug concentrations (0.003%, 0.005%, 0.01%, and 0.1%), frequency of tacrolimus use across different studies, scoring indications, follow-up time, and treatment duration. This variation leads to difficulty in analyzing data and reduces the credibility of the meta-analysis. Shoughy et al. [30] used 0.01% topical tacrolimus, and the frequency was twice daily. However, when a concentration of 0.005% was used by Kheirkhah et al. [15], the frequency was four times daily with safety and efficacy in both studies, but the long-term outcome was not studied. In our study, tacrolimus skin ointment 0.03% was also used twice daily, which showed clinical efficacy and safety in the long term. The efficacy and safety of tacrolimus eye drop in different concentrations (0.003%, 0.005%, 0.01%, and 0.1%) and 0.03% skin ointment for the treatment of VKC have been reported. It is effective in reducing clinical signs and symptoms of severe VKC that are refractory to topical antihistamines and topical cyclosporin [7-11].

There are certain limitations in our study. First, it is a prospective study with a lack of a control group. Second, selection bias was possible as we considered only those patients who could be followed up for two years. Third, non-responders were excluded from the study, and there is a need to study those cases. Thus, further randomized control trials are needed to determine proper dosage, frequency of administration, and scoring indications in clinical studies to facilitate data analysis.

## Conclusions

It can be concluded that tacrolimus is effective in the long-term management of VKC and does not lead to the complications observed in steroid-based therapy. Irrespective of the severity of the disease, it can be prescribed, resulting in stable stages. With the recurrence of the disease, it can be prescribed to achieve reduction in clinical signs and symptoms. As VKC improves the overall quality of life of patients, protocols should be developed to incorporate tacrolimus into the mainstream management plan of VKC.
